# A case of tuberous sclerosis complex revealed by epilepsy

**DOI:** 10.11604/pamj.2017.28.292.13635

**Published:** 2017-12-05

**Authors:** Noukhoum Koné, Mohamedou Ould Elhousseine

**Affiliations:** 1 Service de Neurochirurgie, Centre Hospitalier de Kiffa, Mauritanie; 2 Service de Pédiatrie, Centre Hospitalier de Kiffa, Mauritanie

**Keywords:** Tuberous sclerois complex, epilepsy, skin lesions

## Image in medicine

Tuberous sclerosis complex (TSC) is a multisystem genetic disease. Its incidence is estimated from 1/5800 to 1/1000 births. It results from the mutation of the TSC1 gene on chromosome 9 which codes for hamartin or the mutation of the TSC2 gene on chromosome 16 which codes for tuberin. In 80% of cases, it is linked to a neo-mutation and in 20% of cases it is autosomal dominant inherited from one of the parents. It is a multisystemic disease characterized by the presence of dysplasias, hamartomas and neoplasias in various organs: skin, kidneys, eyes, heart, lungs and mainly the brain. Clinically, epilepsy is the major complication of TSC and approximately 80-90% of patients will develop epilepsy in their lifetime. These epilepsies aren't specific but are generally characterized during evolution by their phamaco-resistance or by the complexity of the seizures. We report the case of a 12-year-old woman with partial epilepsy secondarily generalized, mental retardation and progressive facial angiofibromas (A) for 7 years. The electroencephalogram (EEG) performed 1 year ago showed bihemispheric irritant signs predominant at the frontotemporal right. The brain CT Scan with (B) and without injection of iodinated contrast agent (C) shows calcified subependymal nodules with supratentorial seat with bilateral asymmetric involvement and uncalcified cortical tubercles. The addition of the characteristic triad (convulsion, mental retardation and sebaceous adenoma) to the CT data enabled us to conclude a TSC. The evolution of convulsive seizures was significantly favorable under antiepileptic monotherapy (phenobarbital). On the other hand, mental retardation persists and angiofibromas continue to multiply at the facial level.

**Figure 1:**
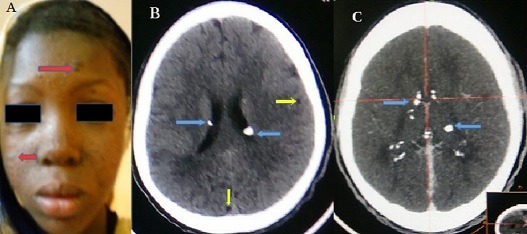
(Red) angiofibromas of the face (A); Brain CT Scan without injection of iodinated contrast agent (B) showing in axial sections calcified subependymal nodules (blue arrow) with supratentorial seat with bilateral asymmetric involvement and uncalcified cortical tubercles yellow arrow) C) Brain CT Scan with injection of iodinated contrast agent

